# Supplementary material for: Using resistor network models to predict the transport properties of solid-state battery composites
Source: Nat Commun. 2025 Feb 6;16:1411. doi: 10.1038/s41467-025-56514-5 (PMC11803108; doi:10.1038/s41467-025-56514-5)
Supplement: Supplementary file 1 — Supplementary Information [file 41467_2025_56514_MOESM1_ESM.pdf]

Supplementary Information –

Using resistor network models to predict the transport properties of solid-state battery composites

Lukas Ketter[a,b], Niklas Greb, Tim Bernges[a], Wolfgang G. Zeier[a,b,c,*]

[a]*Institute of Inorganic and Analytical Chemistry, University of Münster, 48149 Münster, Germany*

[b]*International Graduate School of Battery Chemistry, Characterization, Analysis, Recycling and Application (BACCARA), University of Münster, 48149 Münster, Germany*

[c]*Forschungszentrum Jülich GmbH, Institute of Energy and Climate Research Helmholtz-Institute Münster (IEK-12), 52425 Jülich, Germany*

**Corresponding author email: wzeier@uni-muenster.de*

S1 - Resistor network modelling

S1.1 - Voxel structure generation

Voxel structures are generated by inserting clusters representing the dispersed medium into a matrix representing the continuous medium. Three input parameters, namely the length of the total structure in voxels (L_{vox}), the volume fraction of the dispersed phase (φ_{disp}) in percent and the size of each cluster (s_{clust}) in voxels are required to build the virtual microstructure. The total number (n_{tot}) of voxels is given by $n_{\mathrm{tot}} = L_{\mathrm{vox}}{}^{3}$.

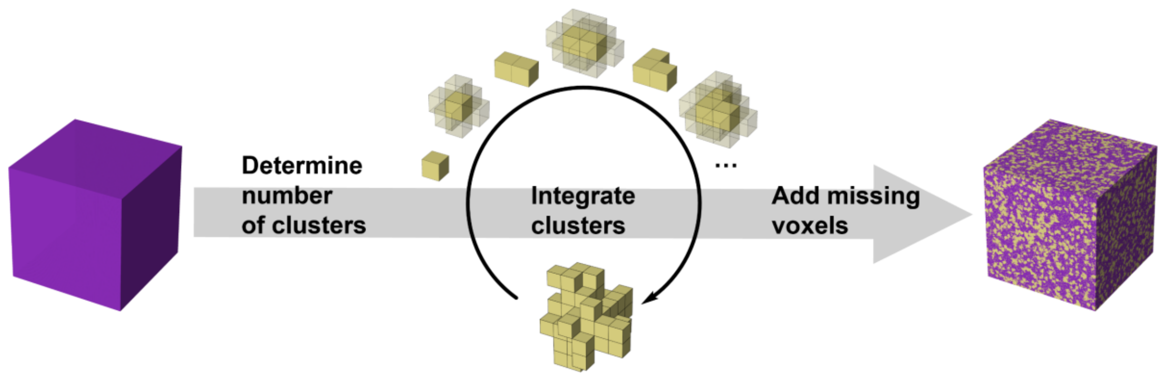

Figure S1: Virtual microstructure generation. *Schematic representation of the algorithm used to generate microstructural models. Voxels colored in purple correspond to the continuous phase, whereas voxels colored in yellow correspond to the dispersed phase.*

Starting from a cubic array filled with voxels representing the continuous medium, clusters of the dispersed medium are iteratively inserted. The number of inserted clusters (n_{clust}) is taken as

$$n_{\mathrm{clust}} = \mathrm{int}\left(\frac{\varphi_{\mathrm{disp}} \cdot n_{\mathrm{tot}}}{100 \cdot s_{\mathrm{clust}}}\right), \qquad \text{(S1)}$$

with int indicating that only the first integer of the expression in brackets is taken as the result. Each cluster is generated by initially choosing a random location in the cubic array. Voxels are then iteratively attached whereby cyclic boundary conditions are applied and new voxels are only allowed to add to the surface of the already existing intermediate cluster (Figure S1). If

the number of clustered voxels equals the desired s_{clust}, the cluster is inserted into the cubic array. Here, overlapping between inserted clusters is permitted. For this reason, and because only the first integer was taken in Eq. S1, the desired volume fraction of the dispersed phase will in many cases not yet be reached. To ensure this nevertheless, the number of voxels missing to reach the desired volume fraction is added without clustering.

S1.2 - Resistor network construction

Subsequently, a resistor network is constructed based on the voxel structure. In a first step, measured conductivities of the continuous (κ_{cont}) and the dispersed phase (κ_{disp}) are assigned to the respective voxels. Then boundary conditions are applied (Figure S2 a). While voxels with infinite conductivity and constant temperature are added to two opposing sides, isolating boundaries are applied to the other surfaces.

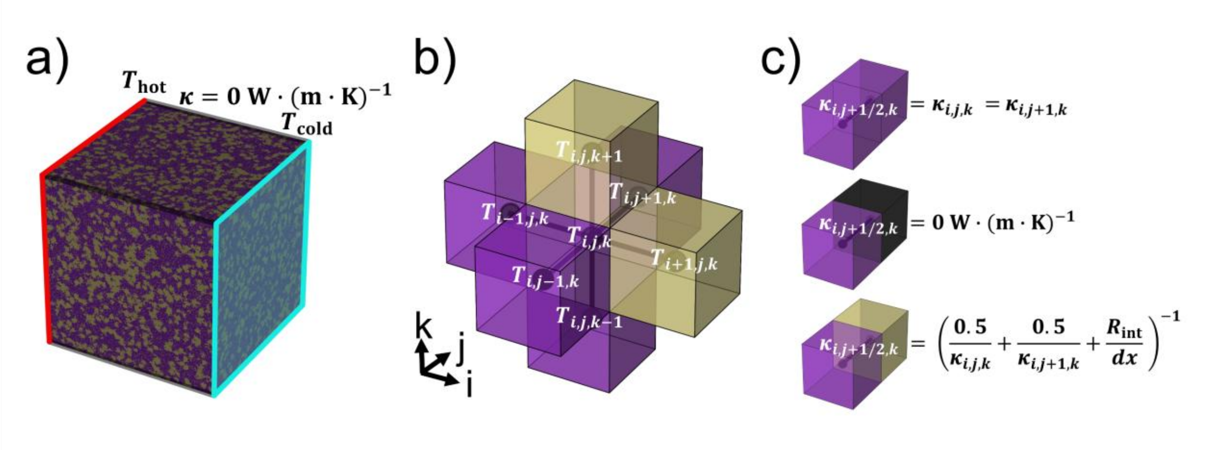

Figure S2: Resistor network construction and boundary conditions. *Voxels colored purple correspond to the continuous phase, whereas voxels colored in yellow represent the dispersed phase. a) Schematic representation of the applied boundary conditions. b) Schematic representation of a voxel with its adjacent neighbors. Each voxel center corresponds to a node in the resistor network. c) Effective conductivities between voxels representing the same material (top), at an isolating boundary (middle) and between voxels representing different materials (bottom).*

Each voxel center corresponds to a node in the resistor network (Figure S2 b). The thermal conductivity between two connected nodes depends on the materials represented by the

voxels. Considering e.g. node $T_{i,j,k}$ and node $T_{i,j+1,k}$ positioned to the back of it (Figure S2 c), the thermal conductivity $\kappa_{i,j+1/2,k}$ of the connection between the nodes is taken as the effective conductivity provided by the series model[1,2]

$$\kappa_{ij+1/2,k} = \left(\frac{0.5}{\kappa_{i,j,k}} + \frac{0.5}{\kappa_{i,j+1,k}}\right)^{-1}. \tag{S2}$$

In the case of a connection between two different materials Eq. S2 is extended to

$$\kappa_{i,j+1/2,k} = \left(\frac{0.5}{\kappa_{i,j,k}} + \frac{0.5}{\kappa_{i,j+1,k}} + \frac{R_{\mathrm{int}}}{dx}\right)^{-1}, \tag{S3}$$

with dx representing the distance between the two nodes.[3] Through this, interfacial resistance (R_{int}) considerations become possible.

S2.3 - Steady state approximation

For better readability, a shorter notation is used in this subchapter:

$T_{\mathrm{spot}} = T_{i,j,k}$, $T_{\mathrm{r}} = T_{i+1,j,k}$, $T_{\mathrm{l}} = T_{i-1,j,k}$, $T_{\mathrm{ba}} = T_{i,j+1,k}$, $T_{\mathrm{f}} = T_{i,j-1,k}$, $T_{\mathrm{t}} = T_{i,j,k+1}$, $T_{\mathrm{b}} = T_{i,j,k-1}$,

$\kappa_{\mathrm{r}} = \kappa_{i+1/2,j,k}$, $\kappa_{\mathrm{l}} = \kappa_{i-1/2,j,k}$, $\kappa_{\mathrm{ba}} = \kappa_{i,j+1/2,k}$, $\kappa_{\mathrm{f}} = \kappa_{i,j-1/2,k}$, $\kappa_{\mathrm{t}} = \kappa_{i,j,k+1/2}$, $\kappa_{\mathrm{b}} = \kappa_{i,j,k-1/2}$.

In the steady state, the total heat flow into and out of each node has to be equal. To meet this condition a temperature distribution fulfilling

$$\begin{aligned} &-\kappa_{\mathrm{r}} \cdot (T_{\mathrm{r}} - T_{\mathrm{spot}}) + \kappa_{\mathrm{l}} \cdot (T_{\mathrm{spot}} - T_{\mathrm{l}}) \\ &-\kappa_{\mathrm{ba}} \cdot (T_{\mathrm{ba}} - T_{\mathrm{spot}}) + \kappa_{\mathrm{f}} \cdot (T_{\mathrm{spot}} - T_{\mathrm{f}}) \\ &-\kappa_{\mathrm{t}} \cdot (T_{\mathrm{t}} - T_{\mathrm{spot}}) + \kappa_{\mathrm{b}} \cdot (T_{\mathrm{spot}} - T_{\mathrm{b}}) = 0 \end{aligned} \tag{S4}$$

for each node is calculated.[1,2] Since this requires solving a large system of linear equations, the iterative successive overrelaxation (SOR) method is implemented. Solving Eq. S4 for T_{spot} results in

$$T_{\mathrm{spot}} = \frac{\kappa_{\mathrm{r}} T_{\mathrm{r}} + \kappa_{\mathrm{l}} T_{\mathrm{l}} + \kappa_{\mathrm{ba}} T_{\mathrm{ba}} + \kappa_{\mathrm{f}} T_{\mathrm{f}} + \kappa_{\mathrm{t}} T_{\mathrm{t}} + \kappa_{\mathrm{b}} T_{\mathrm{b}}}{\kappa_{\mathrm{sum}}} \tag{S5}$$

with $\kappa_{\mathrm{sum}} = \kappa_{\mathrm{r}} + \kappa_{\mathrm{l}} + \kappa_{\mathrm{ba}} + \kappa_{\mathrm{f}} + \kappa_{\mathrm{t}} + \kappa_{\mathrm{b}}$.[1,2] In each iteration, Eq. S5 is solved successively for every node. Taking the temperature thus calculated as $T_{\mathrm{spot}}^{\mathrm{n}}$ and the temperature of the previous iteration $T_{\mathrm{spot}}^{\mathrm{n-1}}$, a new temperature $T_{\mathrm{spot}}^{\mathrm{n,SOR}}$ is calculated via

$$T_{\mathrm{spot}}^{\mathrm{n,SOR}} = T_{\mathrm{spot}}^{\mathrm{n-1}} + \omega\left(T_{\mathrm{spot}}^{\mathrm{n}} - T_{\mathrm{spot}}^{\mathrm{n-1}}\right), \tag{S6}$$

with the relaxation parameter ω.[1,2] As recommended previously, $\omega = 1.979$ was used for structures consisting of 300 x 300 x 300 voxels.[1] The Temperature $T_{\mathrm{spot}}^{\mathrm{n,SOR}}$ is immediately updated as the new node temperature and hence already plays a role when updating the next node. To reduce the number of iterations required to approximate the steady state, the node temperatures are initialized with a linear Temperature distribution. In order to quantify how close a temperature distribution is to the steady state, a residual value (r) is introduced as[2]

$$r = \frac{1}{n_{\mathrm{tot}}} \sum_{i,j,k=0}^{L_{\mathrm{vox}}-1} \left| \frac{\kappa_{\mathrm{sum}} T_{\mathrm{spot}} - \kappa_{\mathrm{r}} T_{\mathrm{r}} - \kappa_{\mathrm{l}} T_{\mathrm{l}} - \kappa_{\mathrm{ba}} T_{\mathrm{ba}} - \kappa_{\mathrm{f}} T_{\mathrm{f}} - \kappa_{\mathrm{t}} T_{\mathrm{t}} - \kappa_{\mathrm{b}} T_{\mathrm{b}}}{\kappa_{\mathrm{sum}}} \right|. \tag{S7}$$

The residual value is calculated every 50 iterations. A representative progression of r with the number of iterations is shown in Figure S3 a. Once r falls below a value of 10^{-9} K the temperature distribution is assumed to be sufficiently close to the steady state and the overall effective conductivity is determined using Fourier's law (Eq. 1). However, for cases where the conductivities of the dispersed and continuous phases are very different and structures near and at the percolation threshold are calculated, the residual value progresses only very slowly with iteration. For a few exceptional cases in this work, the steady state was then assumed to be sufficiently met for $r < 5 \cdot 10^{-9}$ K. Since the effective conductivities of composites at the percolation threshold change dramatically with volume fraction and can reach very low values, these effective conductivities should be treated with caution.

S1.4 - Model validation

To validate the code, effective conductivities were calculated for the trivial cases of series and parallel connection. For these cases, the composition-dependent effective conductivities are given as

$$\kappa_{\text{eff}} = \varphi_1 \cdot \kappa_1 + \varphi_2 \cdot \kappa_2 \quad \text{(S8)}$$

for the parallel model and

$$\kappa_{\text{eff}} = \left(\frac{\varphi_1}{\kappa_1} + \frac{\varphi_2}{\kappa_2}\right)^{-1} \quad \text{(S9)}$$

for the series model.[4]

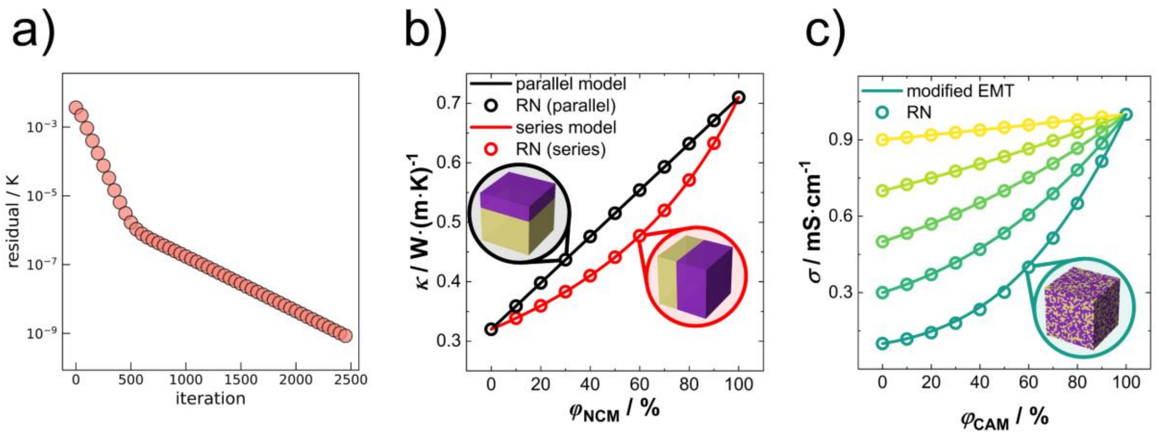

Figure S3: Code validation with analytical solutions. *Voxels colored in purple correspond to the cathode active material phase, whereas voxels colored in yellow correspond to the solid electrolyte phase. a) A representative progression of the residual value with the number of iterations. b) Effective conductivities expected from the analytical solutions for the series and parallel model (lines), compared to effective conductivities simulated using resistor networks representing in parallel and in series connected materials respectively (circles). c) Modified effective medium theory reported by Yuge (lines),[5] compared to effective conductivities calculated using our Resistor network model (circles) with different conductivities of solid electrolyte and cathode active material (CAM). No clustering has been applied when constructing the 30 x 30 x 30 voxel structures to match the conditions described by Yuge.[5]*

Voxel structures representing series and parallel connected materials were constructed and resistor network simulations were performed by using the experimentally assessed thermal conductivities of LPSCl and NCM83 and a thermal interfacial resistance of 0 m^2 K W^{-1} as inputs (Figure S3 b). The simulated effective conductivities agree well with the ones expected from Eq. S8 and Eq. S9.

To further validate the code and to highlight its ability to reliably predict effective composite conductivities when conductivities of e.g. cathode active material and solid electrolyte are close to each other, numerical results by Yuge have been reproduced using the resistor network presented in this work and are shown in Figure S3 c.[5] Voxel structures were constructed using L_{vox} = 30 and s_{clust} = 1, while the conductivities have been varied according to the conditions reported by Yuge.[5] The author also derived a modified effective medium theory (EMT) for conductivities of binary composites on a simple cubic lattice:[5]

$$\sigma_{\mathrm{eff}} = \frac{1}{4} \cdot \Big[(3\varphi_1 - 1)\sigma_{\mathrm{m1}} + (3\varphi_2 - 1)\sigma_{\mathrm{m2}} + \sqrt{[(3\varphi_1 - 1)\sigma_{\mathrm{m1}} + (3\varphi_2 - 1)\sigma_{\mathrm{m2}}]^2 + 8\sigma_{\mathrm{m1}}\sigma_{\mathrm{m2}}}\Big] \tag{S10}$$

with $\sigma_{\mathrm{m1}} = \varphi_1\sigma_1 + \varphi_2 \cdot \frac{2\sigma_1\sigma_2}{\sigma_1+\sigma_2}$ and $\sigma_{\mathrm{m2}} = \varphi_2\sigma_2 + \varphi_1 \cdot \frac{2\sigma_1\sigma_2}{\sigma_1+\sigma_2}$. The numerical results show good agreement with Eq. S10.[5]

S2 - Influence of VGCF additives on the transport properties of solid-state battery composites

In addition to changes in volumetric ratio of SE and CAM on the effective transport properties, the influence of VGCF as electron conducting additive (Figure S4 a) was characterized for a composition of φ_{NCM} = 40 % using the DC-polarization method. A strong increase in the effective electronic conductivity of over two orders of magnitude is observed for VGCF contents above 2 wt.%, while the ionic conductivity is only slightly lowered by introduction of VGCF. Hence, the electronic conductivity can be enhanced almost independently from the ionic transport by VGCF introduction, which is in good agreement with previous findings on VGCF additives in cathode composites.[6]

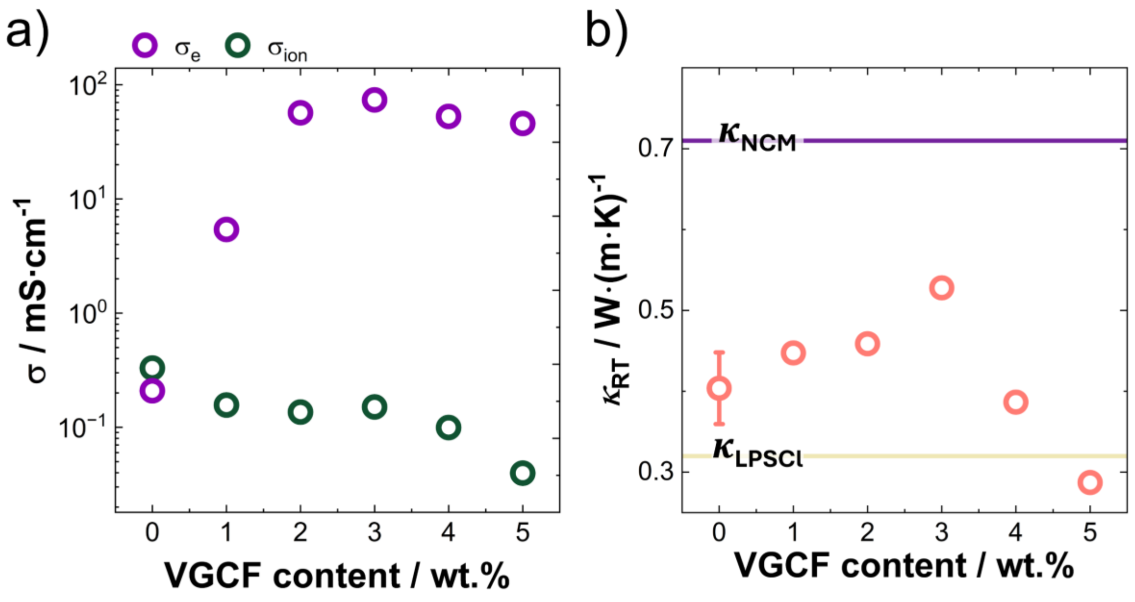

Figure S4: Influence of VGCF on effective transport in NCM83-LPSCl composites. *Effective conductivities as a function of VGCF content in a composite with* φ_{NCM} *= 40 %. a) The effective ionic (green) and electronic (purple) conductivities and b) the effective thermal conductivities as a function of VGCF content. Effective conductivities of LPSCl and NCM83 are shown for comparison in the case of thermal transport. Each data point corresponds to a single measurement, except for the thermal conductivity of the composite with* φ_{NCM} *= 40 % and 0 wt.% VGCF. In this case the thermal conductivity corresponds to the mean value of three*

measurements, whereas the error bars represent the standard deviation of these measurements.

As VGCFs are known to have high thermal conductivity,[7] their influence on the effective thermal conductivity was additionally characterized. Even though VGCFs exhibit remarkably high room temperature thermal conductivities of above 1700 W m^{-1} K^{-1},[7,8] only minor changes in the effective thermal conductivity by VGCF introduction are observed in this work (Figure S4 b). While a slight increase in thermal conductivity is observed for VGCF contents up to 3 wt.%, a drop in geometric density (Figure S18 b) and thermal diffusivity (Figure S16 a) results in a decrease in thermal conductivity for higher carbon contents. The minor influence of VGCF on the effective thermal conductivity could result from the highly anisotropic thermal conductivity properties of the graphitic fibers.[9,10] Furthermore poor adhesion between the fibers and the matrix could ultimately lead to high thermal interfacial resistances between fiber and matrix, explaining the low impact of VGCF introduction on the effective thermal conductivity.[11]

This may indicate that VGCF additives can be used to boost electronic conductivities independently, given that only slightly changes to ion and thermal transport were observed in the investigated composite system.

S3 - Comparison between SEM micrographs, EDX maps and voxel structures

To investigate sample morphologies and homogeneity of LPSCl, NCM83 and NCM83-LPSCl composites, SEM micrographs and EDX maps were measured. Comparisons between the SEM micrographs, EDX mapping results and voxel representations of LPSCl, NCM83 and NCM83-LPSCl composites are shown in Figure S5 and Figure S6.

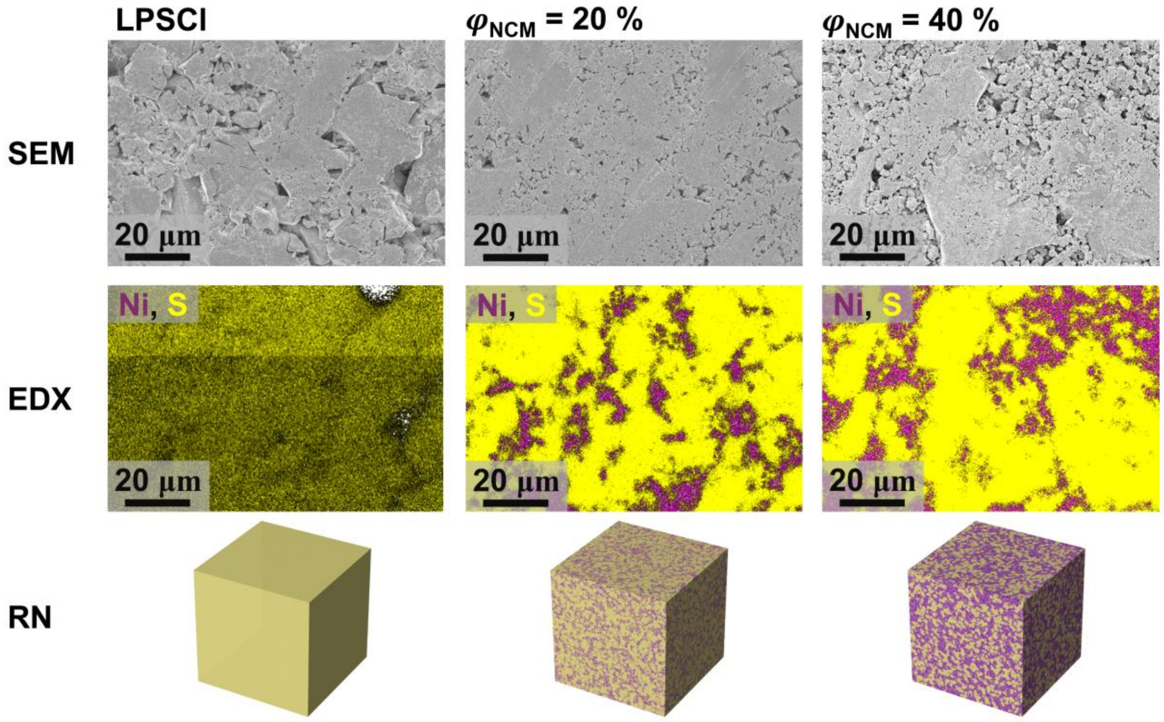

Figure S5: Comparison of SEM micrographs, EDX maps and voxel structures I. *SEM micrographs, overlayed EDX-maps of S (yellow) and Ni (purple) signals and exemplary voxel structures of LPSCl and NCM83-LPSCl composites with* φ_{NCM} *= 20 % and* φ_{NCM} *= 40 %.*

Furthermore, a false-colored SEM micrograph of a NCM83-LPSCl composite with φ_{NCM} = 80 % has been created to highlight the microstructure of that composite. A comparison with an SEM micrograph overlayed with the corresponding EDX-maps of sulfur and nickel is shown in Figure S7.

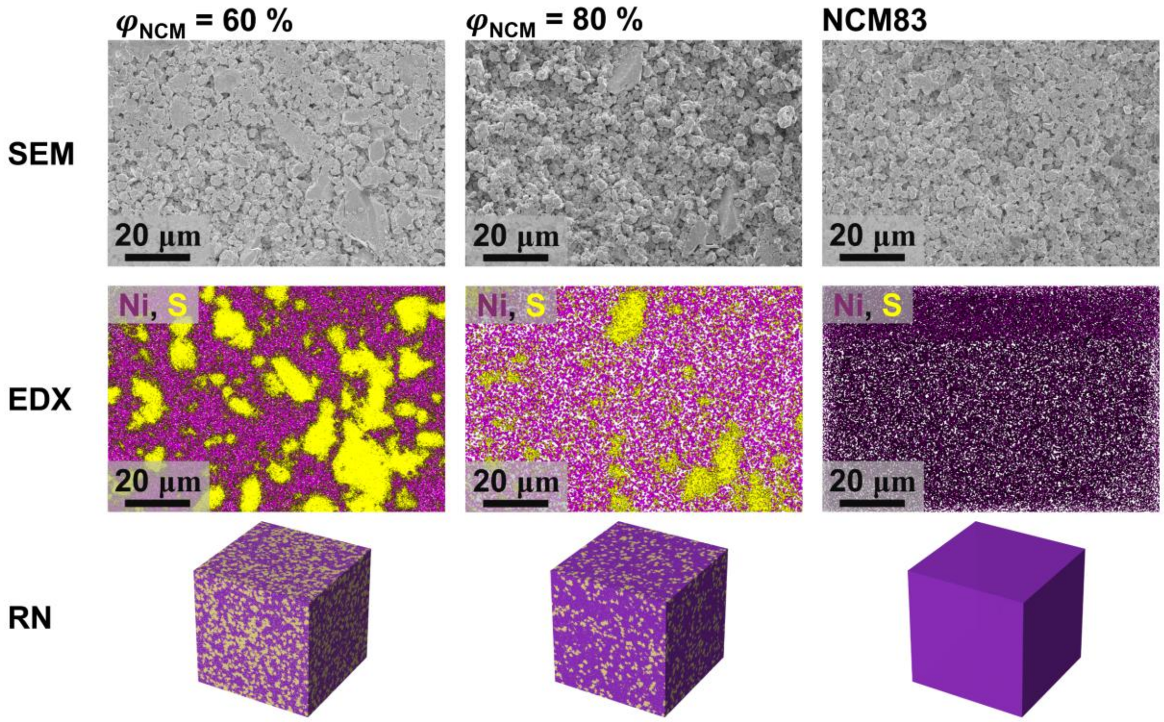

Figure S6: Comparison of SEM micrographs, EDX maps and voxel structures II. *SEM micrographs, overlayed EDX-maps of S (yellow) and Ni (purple) signals and exemplary voxel structures of NCM83-LPSCl composites with* φ_{NCM} *= 60 % and* φ_{NCM} *= 80 % as well as NCM83.*

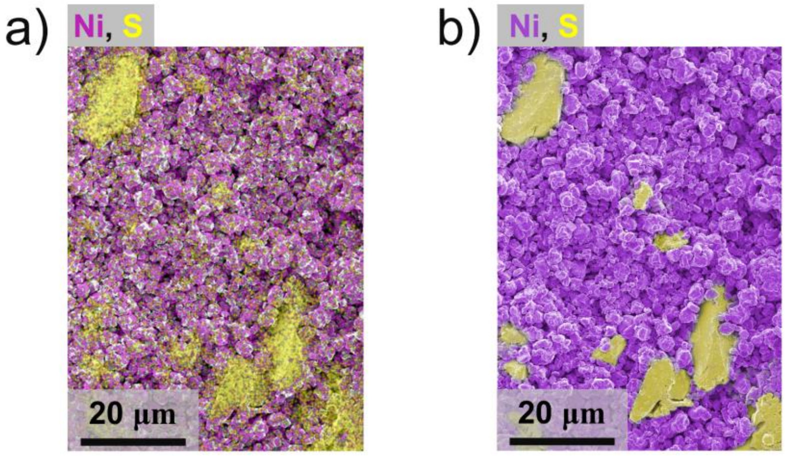

Figure S7: Comparison between an EDX-overlayed SEM micrograph and a corresponding false-colored SEM. *Comparison between a) a SEM micrograph of a NCM83-LPSCl composite with* φ_{NCM} *= 80 % overlayed with the corresponding EDX maps of Sulfur (yellow) and Nickel (purple) and b) the corresponding false-colored SEM micrograph.*

S4 – Electronic Transport measurements

S4.1 - Solid electrolyte Impedance

The impedance data of LPSCl measured at high frequencies are shown in Figure S8 a and can be described by a straight line. As the intercept of the measured impedance corresponds to the bulk resistance of the solid electrolyte,[12] data near this intercept have been fitted by a series-connected resistor and constant phase element (CPE) equivalent circuit to obtain the bulk resistance. Resistance (R) and conductivity (σ) are related via $\sigma = \frac{h}{A \cdot R}$ with sample height (h) and Area (A). With that, determined resistances are converted into conductivities.

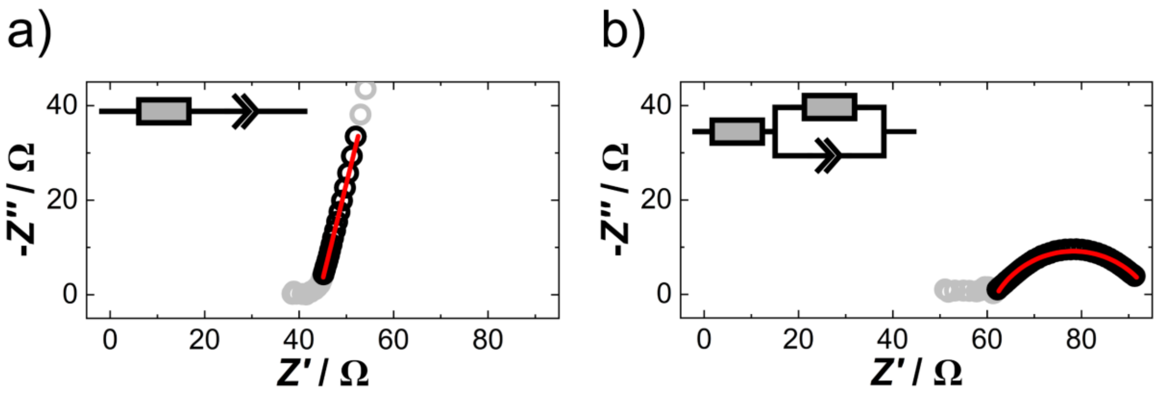

Figure S8: Impedance data of LPSCl measured with different contacts. *Impedance data and fit for a) LPSCl in contact with steel electrodes at high frequencies and b) LPSCl in contact with LiIn alloys. The equivalent circuits used for fitting is shown in the upper left of the respective plots.*

Since LPSCl is practically electronically insulating,[13] the bulk conductivity is assumed as the ionic conductivity of LPSCl. In this work, a room temperature ionic conductivity of 2.33 mS cm^{-1} was determined for LPSCl, which is within the typical range for this electrolyte.[12]

S4.2 - Composite impedance

Transmission line models are widely used as equivalent circuits to understand impedance spectra of composite electrodes.[14]

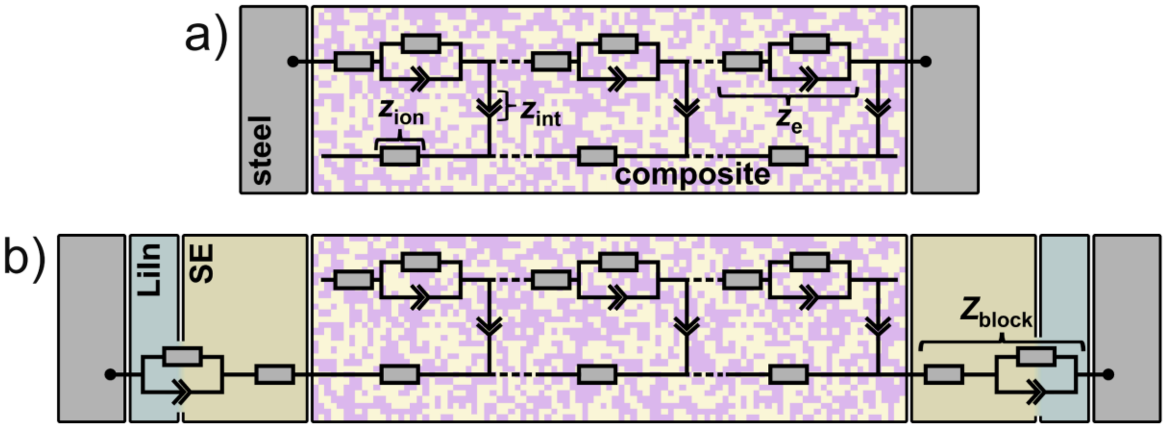

Figure S9: Transmission line models used to evaluate the impedance data of NCM83-LPSCl composites. *Schematic setups and the respective transmission line models employed to fit measured impedance data in an a) ion-blocking and b) electron-blocking measurement setup.*

A T-type transmission line model (TLM), previously described by Siroma *et al.*[15] and reported by Minnmann *et al.*[16] to fit impedances of NCM622-LPSCl cathode composites, is employed in this work to fit impedance data of the closely related NCM83-LPSCl composites (Figure S9). This TLM is a ladder-like equivalent circuit consisting of an infinite number of circuit elements and describes the composite electrode as a continuous medium. The charge carrier transport is modeled by an interconnected ion- and an electron conducting path. Each path consists of infinitely many circuit elements representing ionic (z_{ion}) and electronic (z_{e}) impedances respectively. Also, interfacial impedances (z_{int}) are employed as a connection between the two paths.[15] Depending on the measurement setup, either the ion- or the electron conducting path of the TLM is contacted. In the case of steel electrodes (ion-blocking) the electron conducting path is contacted and the overall impedance (Z_{CC}) is given as

$$Z_{CC} = \frac{z_e z_{ion}}{z_e + z_{ion}} \cdot L + 2 \cdot \frac{{z_e}^2 \sqrt{z_{int}}}{(z_e + z_{ion})^{\frac{3}{2}}} \cdot \frac{\cosh\left(\sqrt{\frac{z_e + z_{ion}}{z_{int}}} \cdot L\right) - 1}{\sinh\left(\sqrt{\frac{z_e + z_{ion}}{z_{int}}} \cdot L\right)}, \quad \text{(S11)}$$

with the electrode length L.[15] While z_{ion} and z_{int} are modeled by a resistor and constant phase element (CPE) respectively, z_e is modeled by a resistor and ZARC element connected in series to account for both, electronic bulk and interfacial charge transport.[16] Multiplying the electrode length with the resistances of z_{ion} and z_e, yields the effective total resistance of the composite to ionic and electronic current respectively.[15] While resistances to electronic current can be resolved well in an ion-blocking measurement setup (Figure S9 a), resistances to ionic current can be resolved well in an electron-blocking measurement setup (Figure S9 b).[16]

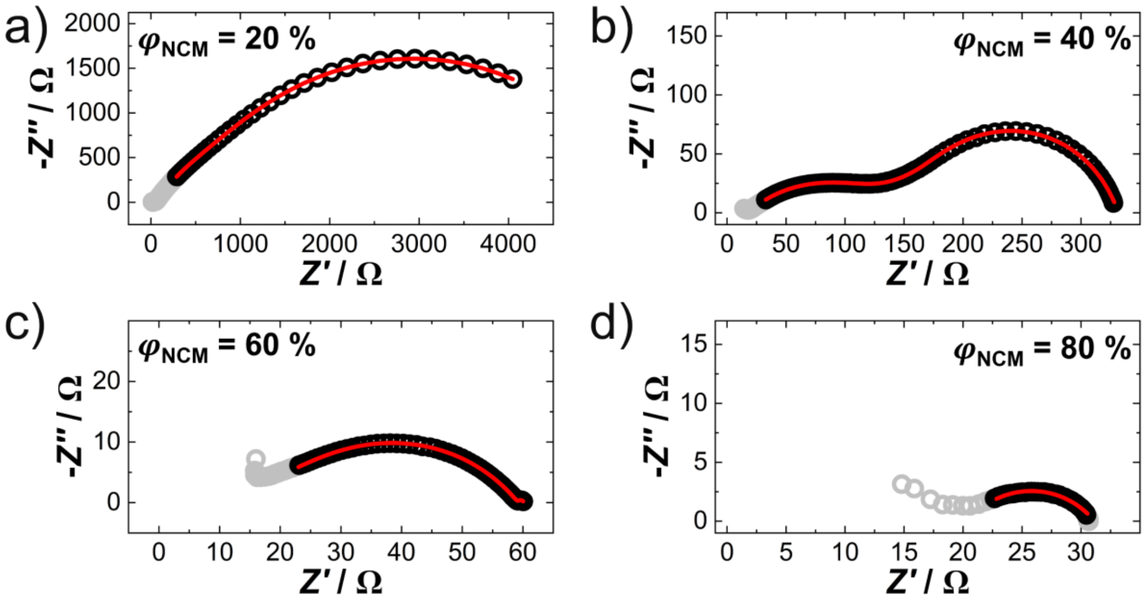

Figure S10: Impedance data of NCM83-LPSCl composites (ion-blocking conditions). *Impedance data and fit of NCM83-LPSCl composites with a)* φ_{NCM} *= 20 %, b)* φ_{NCM} *= 40 %, c)* φ_{NCM} *= 60 %, d)* φ_{NCM} *= 80 %, measured in an ion-blocking setup. A T-type transmission line model was employed to fit the data.*

Since the ion-conducting path is contacted in the latter, z_{ion} and z_e must be swapped in Equation S11 to obtain the correct TLM impedance for this case. As a LiIn alloy and LPSCl layer are employed between the steel electrode and the composite to ensure electron blocking

measurement conditions, additional impedance contributions (Z_{block}) have to be considered through an in-series connection with the TLM. To quantify these contributions a previously reported,[16] symmetric arrangement of LiIn alloy and LPSCl has been measured in this work. The data are shown in Figure S8 b and can be described by a semicircle offset from the origin. The data are fitted using an equivalent circuit consisting of a series-connected resistor and ZARC element. While the resistance corresponds to the bulk resistance of the LPSCl layer, the ZARC element represents the interface between LiIn-alloy and the solid electrolyte.[16]

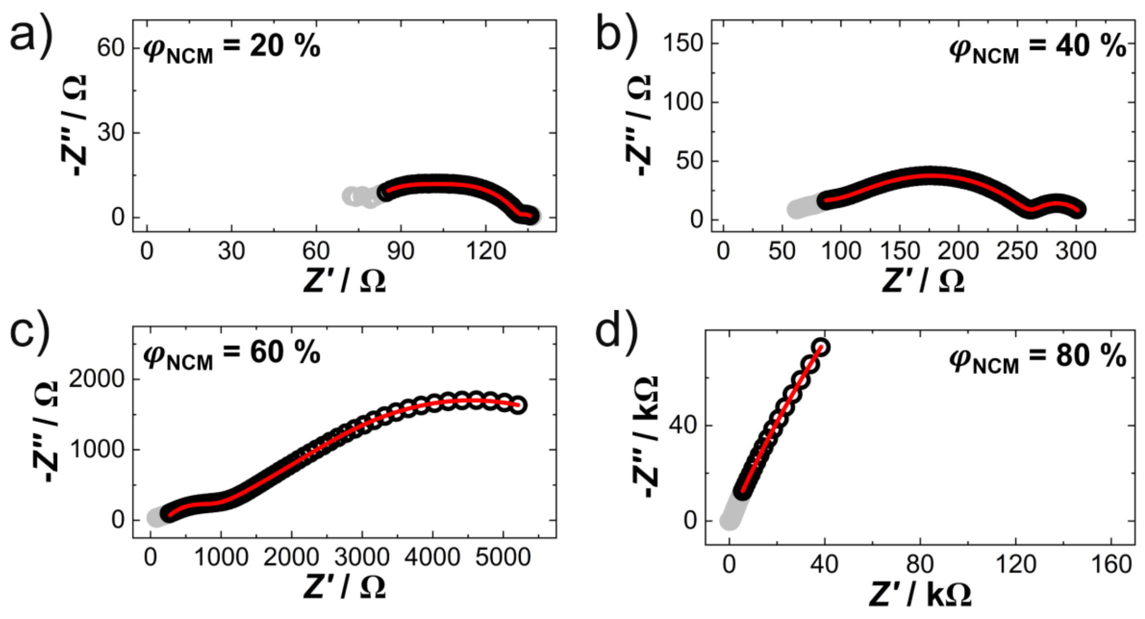

Figure S11: Impedance data of NCM83-LPSCl composites (electron-blocking conditions). *Impedance data and fit of NCM83-LPSCl composites with a) φ_{NCM} = 20 %, b) φ_{NCM} = 40 %, c) φ_{NCM} = 60 %, d) φ_{NCM} = 80 %, measured in an electron-blocking setup. A T-type transmission line model was employed to fit the data.*

Good agreement between impedance data and fits using the T-type TLM model is observed for all composites measured in an ion-blocking (Figure S10) and electron-blocking (Figure S11) measurement setup respectively.

To further verify that the desired effective electronic and ionic conductivities of the composites can be correctly determined by evaluating the impedance data with the TLM, an alternative

equivalent circuit, readily used to evaluate impedance results of mixed ionic-electronic conductors (MIEC) is employed in the following.[17] The model can fit the low frequency impedances of a composite with φ_{NCM} = 40 % (Figure S12). No significant differences in the resulting ionic and electronic conductivities compared to the evaluation with the TLM model are observed, as both circuits yield σ_{e} = 0.36 mS cm^{-1} and σ_{ion} = 0.23 mS cm^{-1} for this composition.

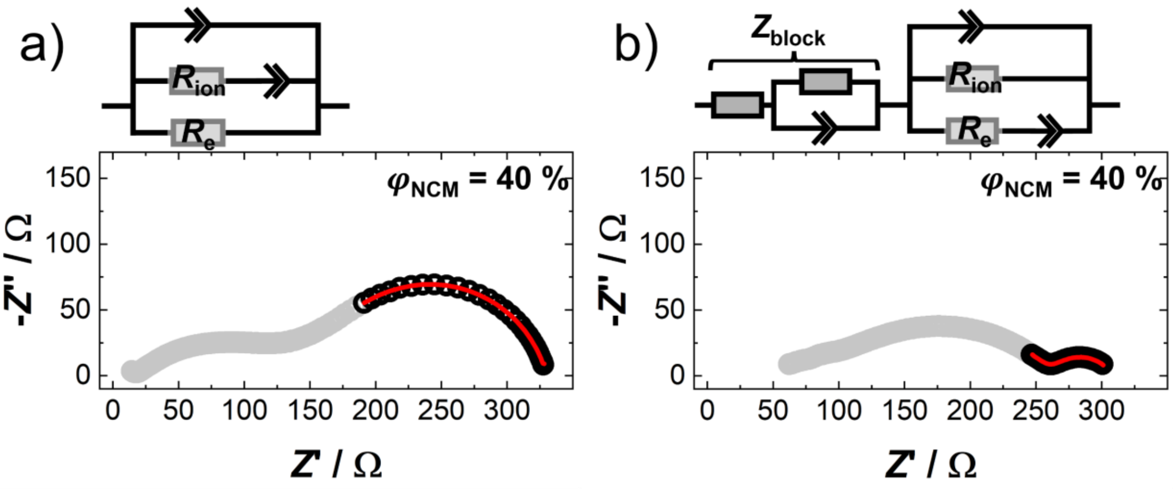

Figure S12: Low frequency impedance modelling using a simplified equivalent circuit. *Impedance data of a composite with* φ_{NCM} *= 40 % measured in an a) ion-blocking and b) electron-blocking setup. The equivalent circuits used for fitting are shown in the top and commonly used by the mixed ionic-electronic conductor community to evaluate effective transport properties.*[17]

The fit qualities of the analysed impedance spectra are listed in Table S1.

Table S1: The quality of the impedance fits is evaluated using χ^2 which is calculated as the modulus-weighted sum of squared residuals per degree of freedom.

Figure	χ^2	Figure	χ^2
S8 a	$2.8 \cdot 10^{-5}$	S11 a	$1.5 \cdot 10^{-6}$
S8 b	$9.2 \cdot 10^{-6}$	S11 b	$6.0 \cdot 10^{-7}$
S10 a	$1.6 \cdot 10^{-6}$	S11 c	$2.5 \cdot 10^{-4}$
S10 b	$3.1 \cdot 10^{-6}$	S11 d	$6.9 \cdot 10^{-6}$
S10 c	$3.2 \cdot 10^{-6}$	S12 a	$3.0 \cdot 10^{-7}$
S10 d	$5.8 \cdot 10^{-6}$	S12 b	$4.1 \cdot 10^{-7}$

S4.3 - Direct current polarization

To validate the conductivities determined using electrochemical impedance spectroscopy and to measure the conductivities of VGCF-containing composites and NCM83, direct current polarization experiments were conducted. In this technique, the sample is exposed to a series of constant voltages. At each voltage step, a steady state is reached over time and the steady-state current is measured. From this, the total resistance of the system is determined using Ohm's law. Typical currents vs. time measured under ion- and electron blocking conditions respectively, as well as resulting voltage vs. steady-state current profiles are shown in Figure S13.

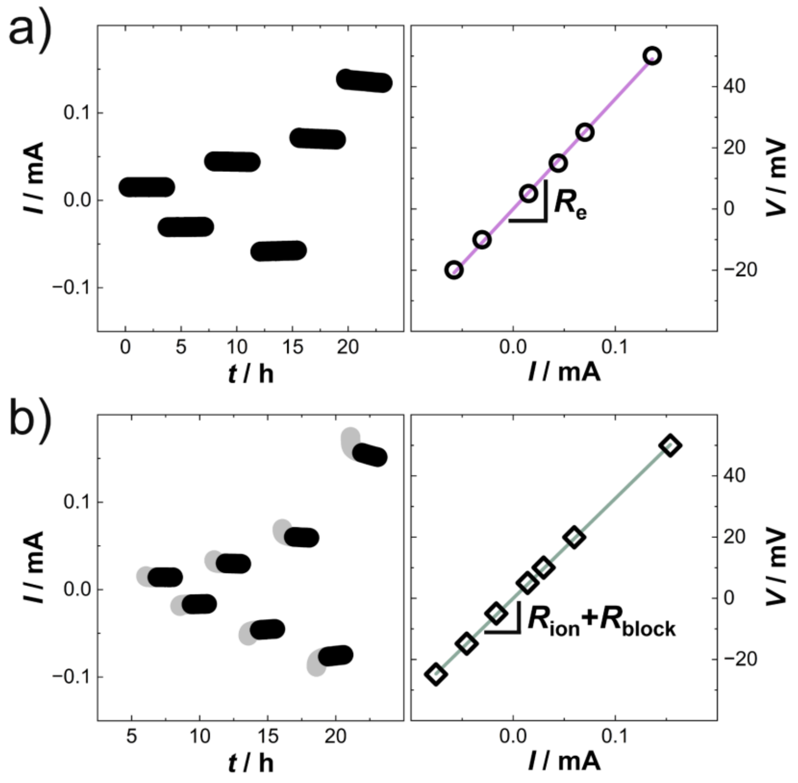

Figure S13: DC polarization experiments – exemplary raw data and analysis. *Measured currents versus time and resulting voltages versus steady-state currents of a NCM83-LSPCl composite with* φ_{NCM} *= 40 % measured under a) ion-blocking and b) electron-blocking conditions.*

Since only electrons contribute to the overall current flow in the steady state under ion-blocking conditions and, conversely, only ions contribute to the total current under electron-blocking conditions, resistance to ion- and electron current can be determined through this method.[18,19] In Figure S14, applied voltages are shown versus the measured steady-state currents for all DC-polarization experiments conducted in this work.

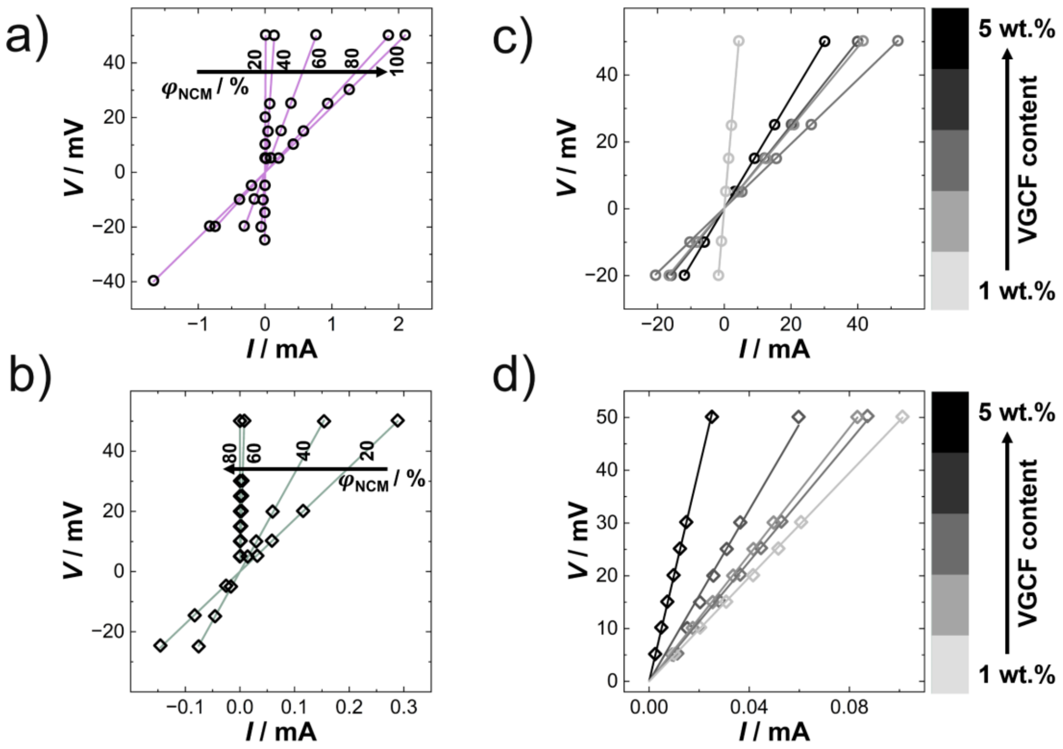

Figure S14: Evaluation of DC polarization experiments. *Applied voltages versus measured steady-state currents, as well as fits with Ohm's law. The slope of each fit corresponds to the total Resistance measured in the steady state. a) measurement results of NCM83, and NCM83-LPSCl composites with* φ_{NCM} *= 20 %,* φ_{NCM} *= 40 %,* φ_{NCM} *= 60 and* φ_{NCM} *= 80 % in an ion-blocking measurement setup and b) in an electron-blocking measurement setup c) measurement results of NCM83-LPSCl-VGCF composites with* φ_{NCM} *= 40 % and VGCF contents from 1 wt.% to 5 wt.% VGCF in an ion-blocking measurement setup and d) in an electron-blocking measurement setup.*

S5 – Temperature dependent thermal diffusivities and thermal conductivities

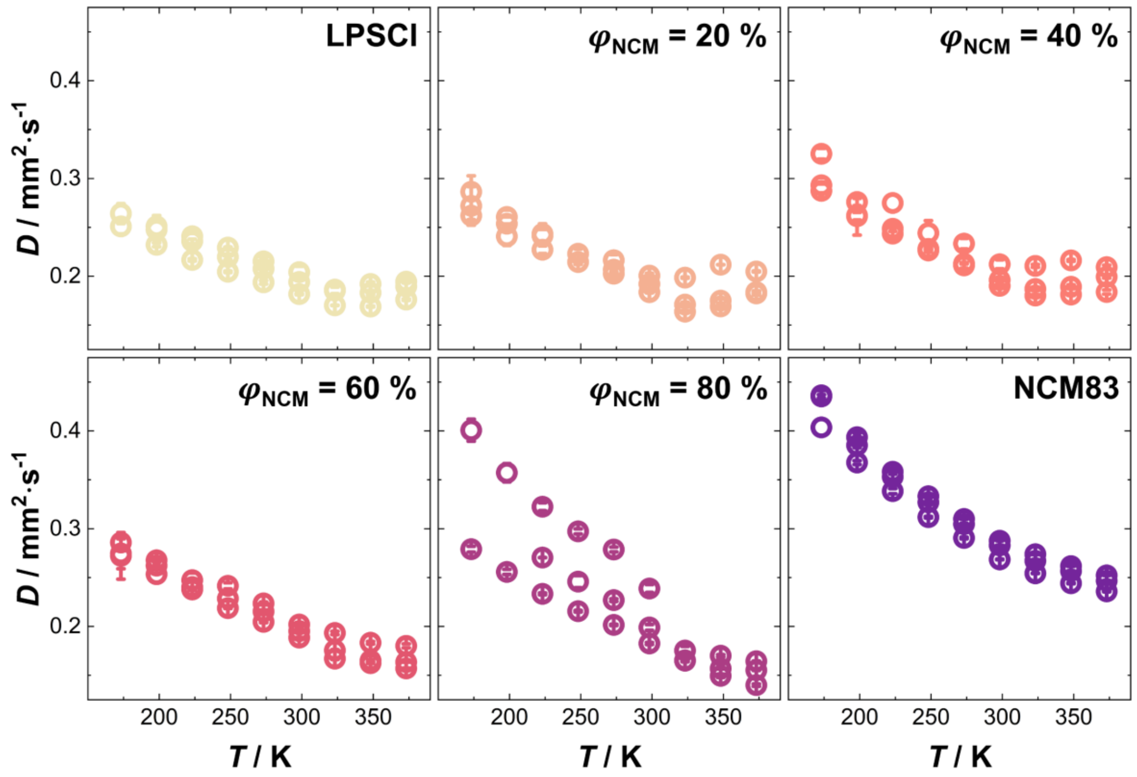

Figure S15: Temperature dependent thermal diffusivities of LPSCl, NCM83 and NCM83-LPSCl composites. *Each data point corresponds to a single measurement, whereas the error bars correspond to the measurement uncertainties.*

Temperature dependent thermal diffusivities and thermal conductivities of VGCF-containing composites

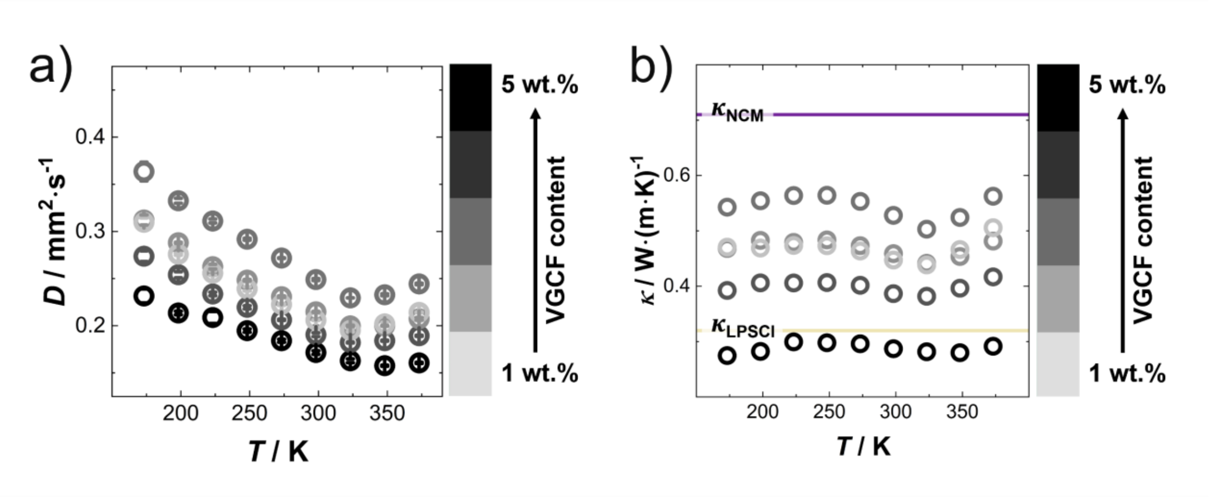

Figure S16: Thermal diffusivities and conductivities of VGCF containing composites. *a) Thermal diffusivities and b) thermal conductivities of NCM83-LPSCl composites with* φ_{NCM} *= 40 % and different amounts of VGCF additives. Each data point corresponds to a single measurement, whereas the error bars represent the measurement uncertainties.*

S6 - Heat capacity calculations

In this work, the isobaric heat capacities of LPSCl and NCM83 are approximated by isochoric heat capacities. While isochoric heat capacities from phonon calculations published by Böger *et al.*[20] were taken for LPSCl, the isochoric heat capacities of NCM83 were calculated based on reported phonon density of states of $\mathrm{Li}M\mathrm{O_2}\,(M = \mathrm{Ni, Mn, Co})$.[21] The normalized phonon density of states ($g(\omega)$) is linked to the specific isochoric heat capacity by

$$C_V = \frac{3 \cdot z \cdot R}{M} \cdot \int_0^\infty g(\omega) \cdot \left(\frac{\hbar\omega}{k_B T}\right)^2 \cdot \frac{\mathrm{e}^{\frac{\hbar\omega}{k_B T}}}{\left(\mathrm{e}^{\frac{\hbar\omega}{k_B T}} - 1\right)^2} d\omega, \tag{S12}$$

with the number of atoms per chemical formula (z), the molar mass (M) and the angular frequency (ω).[22] Through this, isochoric heat capacities of $\mathrm{Li}M\mathrm{O_2}\,(M = \mathrm{Ni, Mn, Co})$ are calculated. The overall specific heat capacity of $\mathrm{LiNi_{0.83}Co_{0.11}Mn_{0.06}O_2}$ is then approximated as $C_{V,\mathrm{NCM83}} \approx\ 0.83 \cdot C_{V,\mathrm{LiNiO_2}} + 0.11 \cdot C_{V,\mathrm{LiCoO_2}} + 0.06 \cdot C_{V,\mathrm{LiMnO_2}}$ (Figure S17).

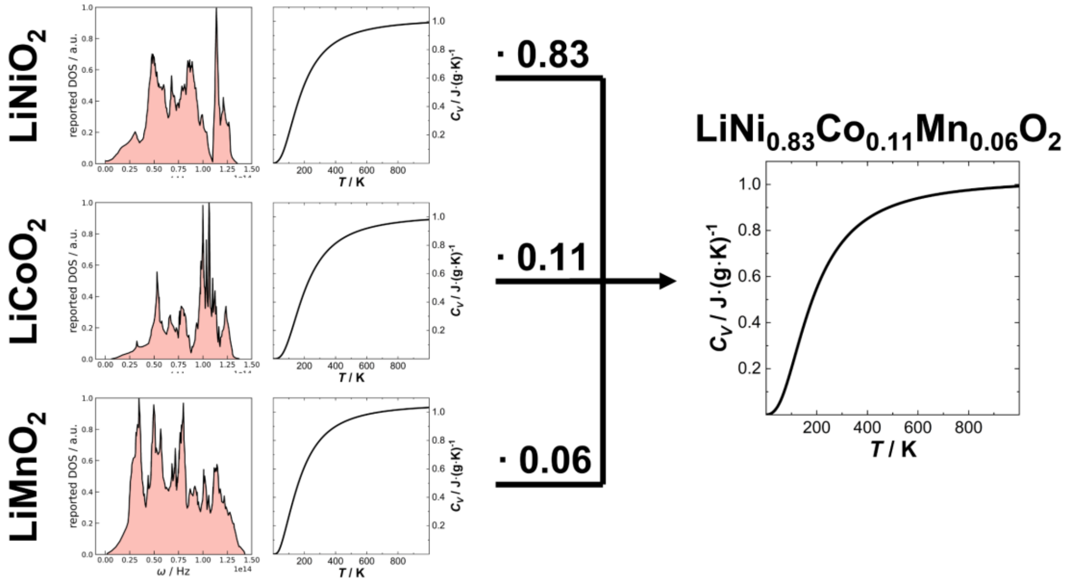

Figure S17: Heat capacity approximation for NCM83. *Left: Literature phonon density of states*[21] *and calculated isochoric heat capacities of $LiMO_2$ $(M = Ni, Mn, Co)$. Right: Combined Heat capacities, used as an approximate for the overall specific heat capacity of $LiNi_{0.83}Co_{0.11}Mn_{0.06}O_2$ (NCM83).*

To obtain the effective heat capacities of the composites, the mass weighted averages[4] are calculated via

$$C_{V,\mathrm{Comp}} = w_{\mathrm{NCM83}} \cdot C_{V,\mathrm{NCM83}} + w_{\mathrm{LPSCl}} \cdot C_{V,\mathrm{LPSCl}} + w_{\mathrm{VGCF}} \cdot C_{V,\mathrm{VGCF}} \tag{S13}$$

The specific heat capacity of VGCF was taken as 0.7 J g^{-1} K^{-1}.[7] Since the highest VGCF content in this work is 5 wt.%, only a minor influence of VGCF on the total heat capacity is observed.

S7 - Geometrical, theoretical and relative densities

The geometrical densities of NCM83, LPSCl, NCM83-LSPCl composites and VGCF containing samples after isostatic densification at 500 MPa for 60 min are shown in Figure S18. In the NCM83-LPSCl system, the average geometrical sample densities (ρ_{geo}) increase linearly from LPSCl (ρ_{geo} = 1.63 g cm^{-3}) to NCM83 (ρ_{geo} = 3.38 g cm^{-3}). However, the relative densities (ρ_{rel}) show an opposing trend decreasing linearly from LPSCl to NCM83. As the input parameters for simulations with resistor networks were taken from porous samples and a linear relationship between composition and density exists, porosity is accounted for in the simulation to a certain extent, although the actual pore structure is not explicitly simulated.

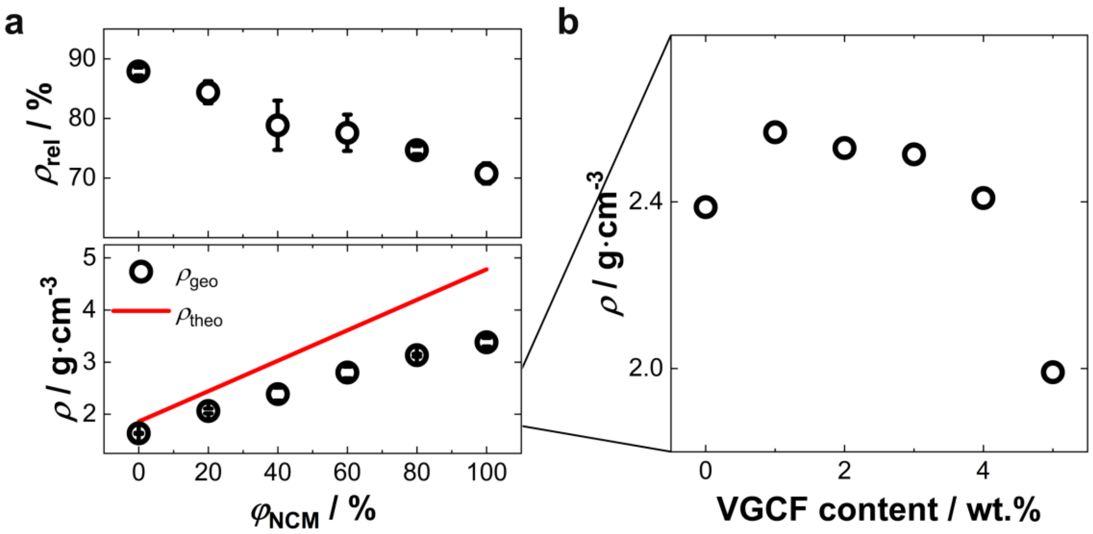

Figure S18: Densities of NCM, LPSCl and composites. *a) Top: Average relative densities of pelletized NCM83-LPSCl composites. Bottom: Theoretical densities[23,24] and geometrical densities of pelletized NCM83-LPSCl composites. The data and error bars correspond to the mean value of three measurements and their standard deviation respectively b) Geometrical densities of pelletized NCM83-LSPCl composites with φ_{NCM} = 40 % and different amounts of VGCF additives. For composites with carbon additives, each data point corresponds to a single measurement.*

The geometrical densities of pelletized NCM83-LPSCl composites with φ_{NCM} = 40 % and VGCF-contents from 1 wt.% to 4 wt.% are similar at ≈ 2.4 g cm^{-3}. However, for a carbon content of 5 wt.%, the sample density is significantly lower.

S8 – XRD patterns before and after the LFA experiment

During the sample transfer into the LFA, the samples are exposed to air for a short time period (< 30 s). As previously shown,[20] this sample transfer does not significantly affect the air-sensitive LPSCl. Nevertheless, to exclude sample degradation during sample transfer and measurement, XRD-patterns were recorded before and after the LFA-experiment and refined using the Pawley-method. The XRD-patterns and Pawley refinements of NCM83-LPSCl composites are shown in Figure S19 and Figure S20.

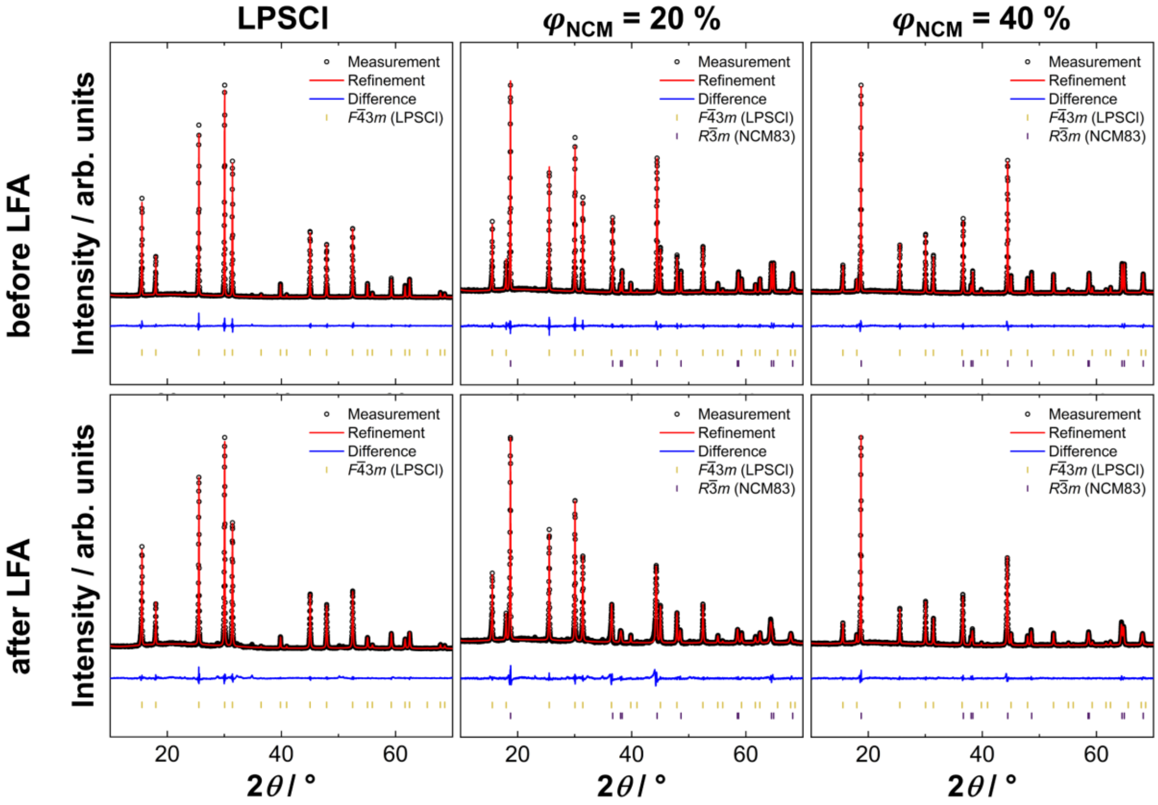

Figure S19: Comparison of XRD patterns before and after the LFA experiment I. *XRD patterns before (top) and after (bottom) the LFA-experiment as well as results of the respective Pawley refinements for LPSCl and NCM83-LPSCl composites with φ_{NCM} = 20 %, φ_{NCM} = 40 %. No side phases were observed after the LFA measurement.*

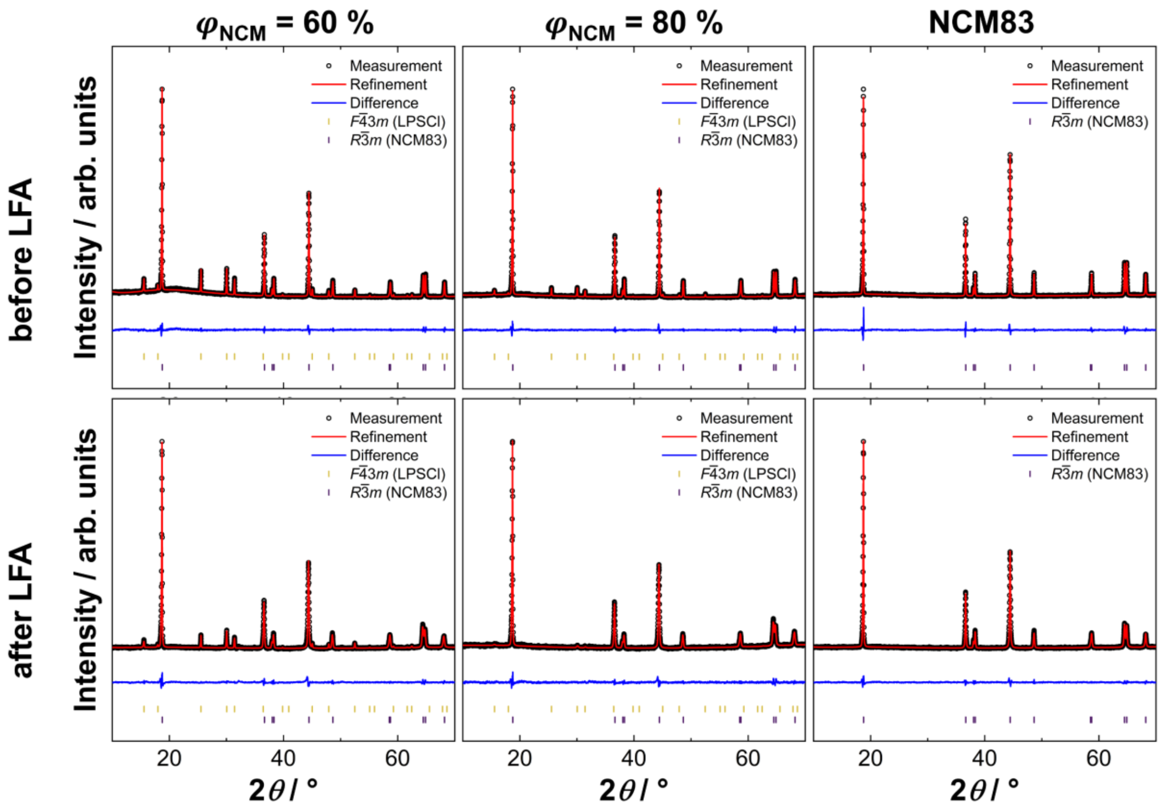

Figure S20: Comparison of XRD patterns before and after the LFA experiment II. *XRD patterns before (top) and after (bottom) the LFA-experiment as well as results of the respective Pawley refinements for NCM83-LPSCl composites with* φ_{NCM} *= 60 %,* φ_{NCM} *= 80 % and NCM83. No side phases were observed after the LFA measurement.*

All XRD-patterns could be refined well with the space groups $R\bar{3}m$ and $F\bar{4}3m$ corresponding to the crystal structures of NCM83 and LPSCl respectively, hence verifying the absence of crystalline side phase formation during sample transfer and LFA measurement. The refined lattice parameters are shown in Table S2.

Table S2: Lattice parameter obtained from Pawley refinements of LPSCl, NCM83 and NCM83-LPSCl composites before and after the LFA experiment.

	**before LFA experiment**			**after LFA experiment**		
	$\boldsymbol{F\bar{4}3m}$	$\boldsymbol{R\bar{3}m}$	$\boldsymbol{R\bar{3}m}$	$\boldsymbol{F\bar{4}3m}$	$\boldsymbol{R\bar{3}m}$	$\boldsymbol{R\bar{3}m}$
	a / Å	a / Å	c / Å	a / Å	a / Å	c / Å
LPSCl	9.85557(7)			9.8545(14)		
φ_{NCM} = 20 %	9.85428(9)	2.87295(3)	14.1933(3)	9.8549(3)	2.88687(9)	14.1978(6)
φ_{NCM} = 40 %	9.8547(11)	2.8727(2)	14.191(2)	9.8551(11)	2.87763(3)	14.2020(14)
φ_{NCM} = 60 %	9.8506(2)	2.87189(3)	14.1859(4)	9.8530(4)	2.87659(5)	14.2019(6)
φ_{NCM} = 80 %	9.8504(3)	2.87184(3)	14.18516(3)	9.849(3)	2.87634(6)	14.1994(7)
NCM83		2.8721(2)	14.1841(14)		2.87287(3)	14.1879(4)

S9 – Listed input parameters and additional case study

Table S3: Input parameters used for resistor network simulations. For each system calculations were performed for different volume fractions of the dispersed phase between 0 % and 100 % with $\Delta\varphi_{disp}$ *= 10 %*

Conductivity input	Figure	L_{vox}	s_{clust}	Continuous phase conductivity	Dispersed phase conductivity	R_{int}
This work (ionic)	3 d	300	500	10^{-100} mS cm^{-1}	2.33 mS cm^{-1}	-
This work (electronic)	3 d	300	500	5.22 mS cm^{-1}	10^{-100} mS cm^{-1}	-
This work (thermal)	4 c	300	500	0.71 W m^{-1} K^{-1}	0.32 W m^{-1} K^{-1}	-
This work (thermal)	4 c	300	500	0.71 W m^{-1} K^{-1}	0.32 W m^{-1} K^{-1}	$2\cdot10^{-6}$ m^2 K W^{-1}
Hendriks et al.[19] (ionic)	5 a	300	500	10^{-100} mS cm^{-1}	0.23 mS cm^{-1}	-
Hendriks et al.[19] (electronic)	5 a	300	500	0.12 mS cm^{-1}	10^{-100} mS cm^{-1}	-
Böger et al.[20] (thermal)	5 b	300	500	0.017 W m^{-1} K^{-1}	0.66 W m^{-1} K^{-1}	-
Froboese et al.[25] (ionic)	5 c	300	10^0	0.619 mS cm^{-1}	10^{-100} mS cm^{-1}	-
Froboese et al.[25] (ionic)	5 c	300	10^1	0.619 mS cm^{-1}	10^{-100} mS cm^{-1}	-
Froboese et al.[25] (ionic)	5 c	300	10^2	0.619 mS cm^{-1}	10^{-100} mS cm^{-1}	-
Froboese et al.[25] (ionic)	5 c	300	10^3	0.619 mS cm^{-1}	10^{-100} mS cm^{-1}	-
Froboese et al.[25] (ionic)	5 c	300	10^4	0.619 mS cm^{-1}	10^{-100} mS cm^{-1}	-
Froboese et al.[25] (ionic)	5 c	300	$5\cdot10^4$	0.619 mS cm^{-1}	10^{-100} mS cm^{-1}	-
Rudel et al.[26] (ionic)	S21	300	1	10^{-100} mS cm^{-1}	2.16 mS cm^{-1}	-
This work (ionic)	S22	60	4	10^{-100} mS cm^{-1}	2.33 mS cm^{-1}	-
This work (electronic)	S22	60	4	5.22 mS cm^{-1}	10^{-100} mS cm^{-1}	-
This work (thermal)	S22	60	4	0.71 W m^{-1} K^{-1}	0.32 W m^{-1} K^{-1}	-
This work (thermal)	S22	60	4	0.71 W m^{-1} K^{-1}	0.32 W m^{-1} K^{-1}	$2\cdot10^{-6}$ m^2 K W^{-1}

Experimental effective ionic conductivities of Si-LPSCl-C composites measured by Rudel *et al.*[26] are shown in Figure S21 and compared to effective conductivities calculated using the resistor network approach. The trend predicted by the resistor networks does not agree with the observed experimental trend. Since distinct domains of SE and active material are assumed in the resistor network model (even without applying clustering when building the microstructure), we assume that this mismatch results from no well-defined microstructure being present in the real composites investigated by Rudel *et al.*[26]

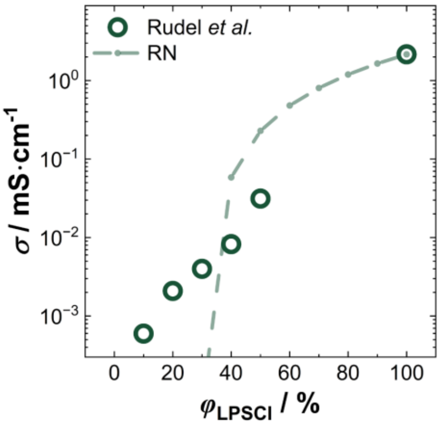

Figure S21: Simulating ion transport of Si-LPSCl-C composites using the resistor network model. *Literature data by Rudel et al.[26] as well as effective ionic conductivities simulated using the resistor network (RN) model.*

S10 – Effect of the total network size on the effective conductivity result

Simulations on small and large resistor networks are compared in Figure S22. While calculations on small resistor networks were performed within less than 1 hour for each composition dependent conductivity series, calculations on large resistor networks come with higher computational costs and require the use of high-performance computing infrastructure. In both cases composite structures with a total edge length of 600 µm are represented by each voxel structure. Since in the case of only using 60 x 60 x 60 voxels, each voxel represents a larger domain of either SE or CAM, in this case a smaller cluster size is chosen when building the virtual composite, to simulate structures with comparable homogeneity. For compositions, far from the percolation threshold, similar effective conductivities are predicted by the small and large resistor network models. As larger relative changes in the effective conductivities show for different microstructures when approaching the percolation threshold (Figure 5 c), stronger deviations between the results of small and large resistor networks are observed here. Comparing the effective simulated thermal conductivities without interfacial resistance, similar results are observed. Introducing the same interfacial thermal resistance for both resistor networks however, a weaker effect is observed for smaller networks. This is probably due to a smaller number of interfaces in the case of the smaller resistor networks. As both, the effective

conductivity near and at the percolation threshold as well as the effect of interfacial thermal resistance are strongly dependent on the real microstructure, both can only be estimated using the resistor network approach and the actual results might differ significantly from the simulated results.

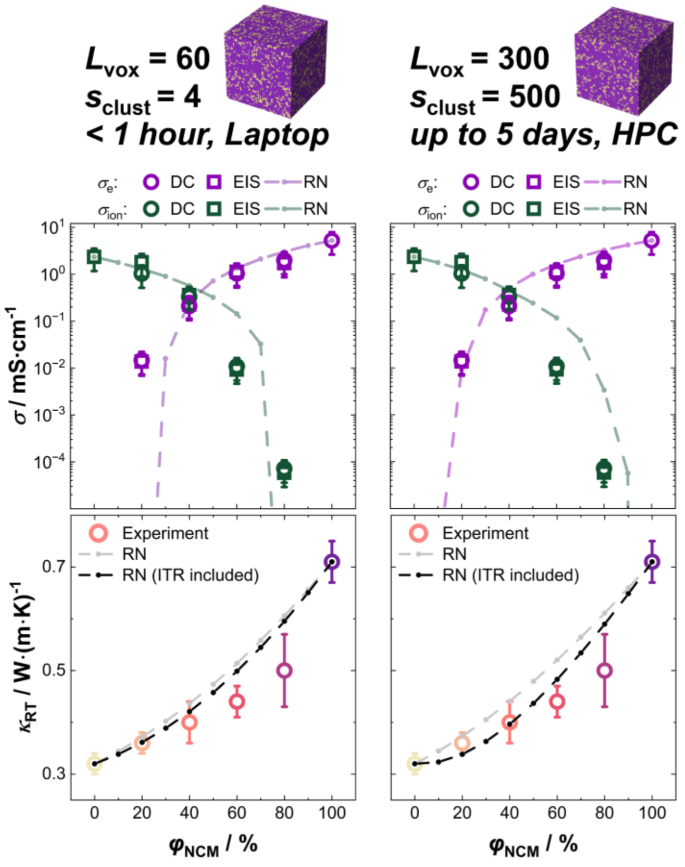

Figure S22: Influence of the microstructure resolution on computation time and quality of the result. *Comparison between simulation results calculated on small (left) and large (right) total voxel structures. A representative voxel structure with* φ_{NCM} *= 80 % is shown for each case. The measured electrical transport data shown corresponds to individual measurements. The error bars correspond to a relative deviation of 50 %. The measured data given for thermal transport corresponds to the average values of three measurements, while the error bars indicate the standard deviations of these measurements.*

S11 – Volume and weight fractions of the investigated composites

The volume ratios (φ_{NCM} / φ_{LPSCl}) and weight ratios (w_{NCM} / w_{LPSCl}) of the investigated NCM83-LPSCl composites are specified in Table S4.

Table S4: Volume fractions and weight fractions of the investigated NCM83-LPSCl composites.

φ_{NCM} / φ_{LPSCl}	100 / 0	80 / 20	60 / 40	40 / 60	20 / 80	0 / 100
w_{NCM} / w_{LPSCl}	100 / 0	91 / 9	79 / 21	63 / 37	39 / 61	0 / 100

Supplementary References

1. Jiang Hao Qiao, Rodolphe Bolot, Hanlin Liao, Pierre Bertrand & Christian Coddet. A 3D finite-difference model for the effective thermal conductivity of thermal barrier coatings produced by plasma spraying. *International Journal of Thermal Sciences* **65,** 120–126; 10.1016/j.ijthermalsci.2012.09.008 (2013).

2. Bolot, R., Antou, G., Montavon, G. & Coddet, C. A Two-Dimensional Heat Transfer Model for Thermal Barrier Coating Average Thermal Conductivity Computation. *Numerical Heat Transfer, Part A: Applications* **47,** 875–898; 10.1080/10407780590921953 (2005).

3. Aalilija, A., Gandin, C.-A. & Hachem, E. A simple and efficient numerical model for thermal contact resistance based on diffuse interface immersed boundary method. *International Journal of Thermal Sciences* **166,** 106817; 10.1016/j.ijthermalsci.2020.106817 (2021).

4. Carson, J. K. Modelling Thermal Diffusivity of Heterogeneous Materials Based on Thermal Diffusivities of Components with Implications for Thermal Diffusivity and Thermal Conductivity Measurement. *Int J Thermophys* **43**; 10.1007/s10765-022-03037-6 (2022).

5. Yuge, Y. Three-dimensional site percolation problem and effective-medium theory: A computer study. *J Stat Phys* **16,** 339–348; 10.1007/BF01020426 (1977).

6. Hayakawa, E., Nakamura, H., Ohsaki, S. & Watano, S. Design of active-material/solid-electrolyte composite particles with conductive additives for all-solid-state lithium-ion batteries. *Journal of Power Sources* **555,** 232379; 10.1016/j.jpowsour.2022.232379 (2023).

7. Ting, J.-M. & Lake, M. L. Vapor-grown carbon-fiber reinforced carbon composites. *Carbon* **33,** 663–667; 10.1016/0008-6223(94)00153-Q (1995).

8. Heremans, J., Rahim, I. & Dresselhaus, M. S. Thermal conductivity and Raman spectra of carbon fibers. *Physical review. B, Condensed matter* **32,** 6742–6747; 10.1103/PhysRevB.32.6742 (1985).

9. Lee, E. *et al.* Design of lithium cobalt oxide electrodes with high thermal conductivity and electrochemical performance using carbon nanotubes and diamond particles. *Carbon* **129,** 702–710; 10.1016/j.carbon.2017.12.061 (2018).

10. Chen, J., Walther, J. H. & Koumoutsakos, P. Covalently Bonded Graphene–Carbon Nanotube Hybrid for High-Performance Thermal Interfaces. *Adv Funct Materials* **25,** 7539–7545; 10.1002/adfm.201501593 (2015).

11. Macedo, F. & Ferreira, J. A. Thermal contact resistance evaluation in polymer-based carbon fiber composites. *Review of Scientific Instruments* **74,** 828–830; 10.1063/1.1520325 (2003).

12. Kraft, M. A. *et al.* Influence of Lattice Polarizability on the Ionic Conductivity in the Lithium Superionic Argyrodites Li6PS5X (X = Cl, Br, I). *Journal of the American Chemical Society* **139,** 10909–10918; 10.1021/jacs.7b06327 (2017).

13. Gorai, P., Famprikis, T., Singh, B., Stevanović, V. & Canepa, P. Devil is in the Defects: Electronic Conductivity in Solid Electrolytes. *Chemistry of Materials* **33,** 7484–7498; 10.1021/acs.chemmater.1c02345 (2021).

14. Siroma, Z. *et al.* AC impedance analysis of ionic and electronic conductivities in electrode mixture layers for an all-solid-state lithium-ion battery. *Journal of Power Sources* **316,** 215–223; 10.1016/j.jpowsour.2016.03.059 (2016).

15. Siroma, Z. *et al.* Mathematical solutions of comprehensive variations of a transmission-line model of the theoretical impedance of porous electrodes. *Electrochimica Acta* **160,** 313–322; 10.1016/j.electacta.2015.02.065 (2015).

16. Minnmann, P., Quillman, L., Burkhardt, S., Richter, F. H. & Janek, J. Editors' Choice—Quantifying the Impact of Charge Transport Bottlenecks in Composite Cathodes of All-Solid-State Batteries. *J. Electrochem. Soc.* **168,** 40537; 10.1149/1945-7111/abf8d7 (2021).

17. Zahnow, J. *et al.* Impedance Analysis of NCM Cathode Materials: Electronic and Ionic Partial Conductivities and the Influence of Microstructure. *ACS Appl. Energy Mater.* **4,** 1335–1345; 10.1021/acsaem.0c02606 (2021).

18. Schlautmann, E. *et al.* Impact of the Solid Electrolyte Particle Size Distribution in Sulfide-Based Solid-State Battery Composites. *Advanced Energy Materials* **13**; 10.1002/aenm.202302309 (2023).

19. Theodoor A. Hendriks, Martin A. Lange, Ellen M. Kiens, Christoph Baeumer & Wolfgang G. Zeier. Balancing Partial Ionic and Electronic Transport for Optimized Cathode Utilization of High-Voltage LiMn2O4/Li3InCl6 Solid-State Batteries. *Batteries and Supercaps* **6**; 10.1002/batt.202200544 (2023).

20. Böger, T., Bernges, T., Li, Y., Canepa, P. & Zeier, W. G. Thermal Conductivities of Lithium-Ion-Conducting Solid Electrolytes. *ACS Appl. Energy Mater.* **6,** 10704–10712; 10.1021/acsaem.3c01977 (2023).

21. Hui Yang *et al.* Chemical Trends in the Lattice Thermal Conductivity of Li(Ni, Mn, Co)O2(NMC) Battery Cathodes. *Chemistry of Materials* **32,** 7542–7550; 10.1021/acs.chemmater.0c02908 (2020).

22. Agne, M. T. *et al.* Heat capacity of Mg3Sb2, Mg3Bi2, and their alloys at high temperature. *Materials Today Physics* **6,** 83–88; 10.1016/j.mtphys.2018.10.001 (2018).

23. Deiseroth, H.-J. *et al.* Li6PS5X: a class of crystalline Li-rich solids with an unusually high Li+ mobility. *Angewandte Chemie (International ed. in English)* **47,** 755–758; 10.1002/anie.200703900 (2008).

24. Woo, S.-W., Myung, S.-T., Bang, H., Kim, D.-W. & Sun, Y.-K. Improvement of electrochemical and thermal properties of Li[Ni0.8Co0.1Mn0.1]O2 positive electrode materials by multiple metal (Al, Mg) substitution. *Electrochimica Acta* **54,** 3851–3856; 10.1016/j.electacta.2009.01.048 (2009).

25. Froboese, L., van der Sichel, J. F., Loellhoeffel, T., Helmers, L. & Kwade, A. Effect of Microstructure on the Ionic Conductivity of an All Solid-State Battery Electrode. *J. Electrochem. Soc.* **166,** A318-A328; 10.1149/2.0601902jes (2019).

26. Rudel, Y. *et al.* Investigating the Influence of the Effective Ionic Transport on the Electrochemical Performance of Si/C-Argyrodite Solid-State Composites. *Batteries and Supercaps* **6**; 10.1002/batt.202300211 (2023).
